# Multidrug-Resistant *Escherichia coli *Isolated from Stool Samples of Healthy Infants in Rural Bangladeshi Communities

**DOI:** 10.4269/ajtmh.24-0541

**Published:** 2025-05-13

**Authors:** Fatema-Tuz Johura, Jarin Tasnim, Sahitya Ranjan Biswas, Riajul Islam, Talal Hossain, Hafizur Rahman, Saijuddin Shaikh, Hasmot Ali, Subhra Chakraborty, Alain Labrique, Tahmeed Ahmed, Md Iqbal Hossain, Munirul Alam, Amanda C. Palmer

**Affiliations:** ^1^ International Centre for Diarrheal Disease Research, Bangladesh, Dhaka, Bangladesh;; ^2^ The JiVitA Research Project, Gaibandha, Bangladesh;; ^3^ Johns Hopkins Bloomberg School of Public Health, Baltimore, Maryland

## Abstract

*Escherichia coli* (*E. coli*) is a commensal organism in humans and animals. It can serve as a reservoir for antibiotic resistance, thus providing an indicator of drug resistance patterns in a community. We investigated antibiotic resistance in *E. coli *isolated from nondiarrheal stool samples of 6-month-old infants (*n* = 110) from northwest Bangladesh. We conducted susceptibility testing using a disc diffusion assay against 20 antibiotics. Resistance was most pronounced for macrolides (98.2% resistant), whereas the most sensitive antibiotics were fosfomycin (100%), gentamicin (99.1%), meropenem (98.2%), mecillinam (97.3%), tigecycline (97.3%), and imipenem (87.3%). Excluding erythromycin, roughly 55% of isolates were multidrug-resistant. Our results likely reflect the burden of drug-resistant *E. coli* in the guts of infants in rural Bangladesh and the prevailing drug resistance patterns in this community.

## INTRODUCTION

Global concern over antibiotic resistance is escalating, and the spread of multidrug-resistant (MDR) strains is a global public health challenge.^1^ Resistant strains of various bacteria are prevalent not only in hospital settings but also in communities. In community settings, infants and young children are often the most exposed to antibiotics, and several studies have indicated that these age groups have the highest risk of carrying antibiotic-resistant commensal bacteria.^2^^–^^5^

*Escherichia coli *(*E. coli*) is a nearly ubiquitous colonizer of the gastrointestinal tract in warm-blooded animals, including humans.^6^ *Escherichia coli* may play a role in the emergence and spread of resistance within a community because fecal flora serve as reservoirs for antibiotic resistance genes.^6^^,^^7^ Exposure of commensals such as *E. coli *to antibiotics increases the carriage levels of resistant organisms, and resistance might be transmitted to more virulent acquired organisms.^8^ Animals, humans, and the environment, including water sources, serve as natural habitats for virulent strains of *E. coli*.^6^^,^^9^

We report on the prevalence of antibiotic resistance in commensal *E. coli *bacteria isolated from stool samples of healthy infants in a community setting in rural Bangladesh.

## MATERIALS AND METHODS

This study was conducted at the JiVitA research site in Gaibandha District in northwestern Bangladesh, which is broadly representative of rural areas.^10^ Samples were collected from May 2019 to March 2020 as part of a cluster-randomized controlled trial (NCT03683667). Infants were identified through a surveillance system and enrolled at ∼6 months of age. Here, we focus on samples collected from a subset of infants (*N* = 489) before the start of the intervention, when the infants ranged in age from 6 to 6.8 months. Samples from the first 125 infants (∼25%) were selected to monitor antibiotic resistance via both *E. coli* isolated from stool samples and *Streptococcus pneumoniae* isolated from nasopharyngeal swabs. This number was increased to 128 samples to account for missing swabs from three infants. Households were provided with a stool collection kit that included a sterile mat, a spoon, a specimen collection cup, a cooler box, and ice packs. Caregivers were instructed to have their infant defecate on the sterile mat, to collect ∼15–20 g of stool into the specimen cup, and to place it in the cooler box. Cooler boxes were retrieved by project staff the following morning. If infants had not yet defecated, two additional attempts were made to collect a stool sample, either in the afternoon or the following night. Cooler boxes were transported to the project laboratory within 8 hours of sample collection, where ∼1 g of stool was aliquoted into a cryovial containing 20% glycerol and stored at −80°C. Samples were transported at 4–6°C to the International Centre for Diarrheal Disease Research, Bangladesh, in Dhaka and stored at −80°C. For processing, a loopful of stool was inoculated directly onto a MacConkey agar plate (Becton, Dickinson and Company, Franklin Lakes, NJ) and incubated at 37°C for 18–24 hours. From each sample, one lactose-fermenting bright pink colony was presumptively identified as *E. coli *based on colony morphology and was further confirmed via biochemical analysis using API 20E (Biomerieux, Marcy-l’Étoile, France). All isolates were stored at −80°C for further analysis.

Each isolate was subjected to antibiotic susceptibility testing against 20 different antibiotics using the Kirby–Bauer disc diffusion method,^11^ according to the Clinical and Laboratory Standards Institute (CLSI) guidelines.^12^ Discs were procured from Oxoid Limited (Hampshire, United Kingdom). The following antibiotics were chosen based on their prevalent local usage^13^^,^^14^ and to cover different chemical classes: ciprofloxacin (5 µg), azithromycin (15 µg), levofloxacin (5 µg), tetracycline (30 µg), trimethoprim–sulfamethoxazole (25 µg), erythromycin (15 µg), ampicillin (10 µg), gentamicin (10 µg), amoxicillin–clavulanate (30 µg), ceftriaxone (30 µg), cefepime (30 µg), cefuroxime (30 µg), meropenem (10 µg), imipenem (10 µg), fosfomycin (50 µg), aztreonam (30 µg), tigecycline (15 µg), mecillinam (25 µg), doxycycline (30 µg), and ceftazidime (30 µg). The resistance and susceptibility profiles of the isolates were determined by measuring the inhibitory zones. *Eschericia coli* reference strain ATCC 25922 was used for quality control.

For each antibiotic, we used cutoffs published by the CLSI^12^ to define whether each isolate was sensitive, intermediate, or resistant. We then calculated the number of antibiotics to which each isolate was resistant. Isolates with resistance to at least one antibiotic in three or more antibiotic classes were classified as MDR. Because no isolates were sensitive to erythromycin, we excluded this antibiotic from our calculations.

## RESULTS

**Figure 1.**
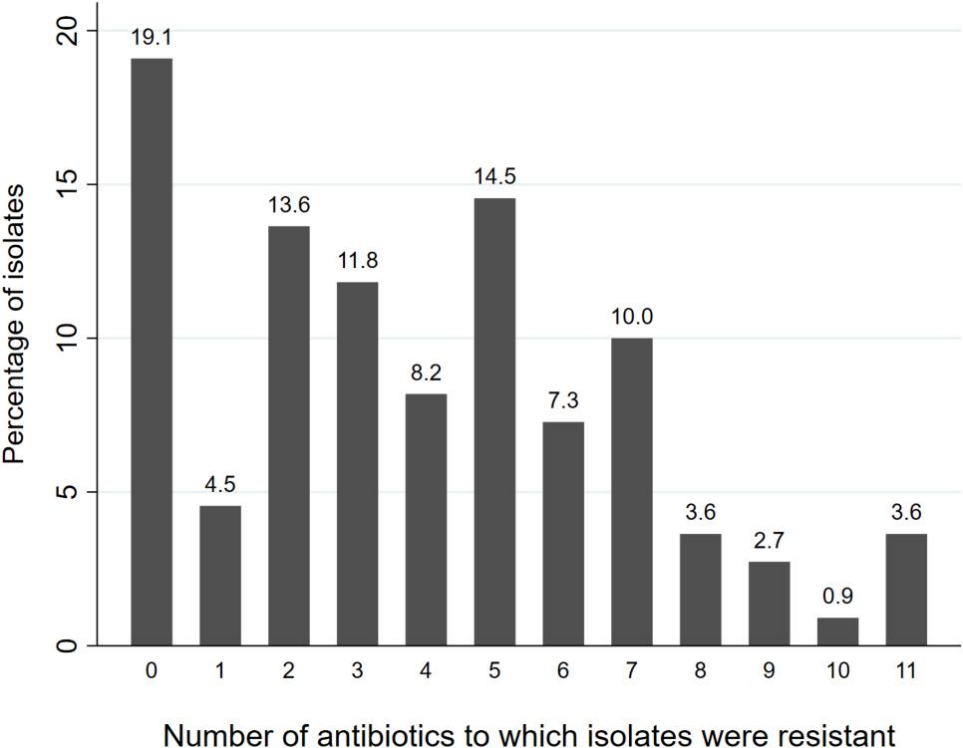
Frequency of resistance phenotypes in antibiotic-resistant *Eschericia coli*. Phenotypes are defined by the number of antibiotics, out of the 19 tested (erythromycin was excluded from this analysis), to which individual isolates were resistant, based on the results of disc diffusion analysis and applying cutoffs from the Clinical and Laboratory Standards Institute.^12^

Of the 489 infants enrolled in the substudy, we collected samples from 401 infants (88 infants did not provide samples after three attempts), and the first 128 were selected for culturing and analysis (Supplemental Figure 1). Households varied in size and occupation (Table 1), with high access to electricity (64.6%), a tubewell (100%), improved latrines (71.7%), and livestock (87.4%). Prevalence estimates for stunting, underweight, and wasting were 14.3%, 9.5%, and 3.2%, respectively. Of the 128 stool samples tested, 110 (86%) yielded *E. coli *using standard culturing methods. Resistance patterns for the 20 antibiotics are shown in Table 2. The highest prevalence of resistance was found for macrolides (98.2% for erythromycin and 63.6% for azithromycin) and for ampicillin (70.0%). There was also an elevated prevalence of resistance (10–34%) to extended-spectrum cephalosporins. The most effective antibiotics were fosfomycin (100% sensitivity), gentamicin (99.1%), meropenem (98.2%), tigecycline (97.3%), mecillinam (97.3%), and imipenem (87.3%). Excluding erythromycin, only 21 (19%) of isolates were sensitive to the 19 antibiotics tested (Figure 1). The median (25th percentile–75th percentile) number of antibiotics to which isolates were resistant was 4 (2–6). A total of 61 isolates (55.5%) were classified as MDR, meaning that they were resistant to at least one antibiotic in three or more antibiotic classes.

**Table 1:** Household and infant characteristics of rural Bangladeshi infants contributing stool samples at 6 months of age (*n* = 128)

Characteristics	Mean ± SD or *n* %
Household characteristics	
Household size	4.5 ± 2.0
Household head’s occupation	
Farmer/sharecropper	13 (11.8)
Laborer	37 (33.6)
Business owner	43 (39.1)
Private service	17 (15.5)
Access to electricity	82 (64.6)
Access to tubewell	127 (100.0)
Access to sanitation	
None/field/bush	4 (3.1)
Open latrine	21 (16.5)
Improved latrine	91 (71.7)
Flush toilet	11 (8.7)
Own any livestock	104 (87.4)
Infant characteristics	
Male sex	61 (47.7)
Age, months	6.4 ± 0.3
Anthropometry	
LAZ	−1.0 ± 1.0
Stunted (LAZ < –2)	18 (14.3)
WAZ	−0.8 ± 1.0
Underweight (WAZ < –2)	12 (9.5)
WLZ	−0.2 ± 1.0
Wasted (WLZ < –2)	4 (3.2)
Breastfed	126 (98.4)
Morbidity, past 7 days	
Diarrhea	0 (0)
Dysentery	2 (1.6)
High fever	17 (13.4)
Cough/cold	52 (40.6)

LAZ = length-for-age Z-score; WAZ = weight-for-age Z-score; WLZ = weight-for-length Z-score.

**Table 2:** Antimicrobial susceptibility pattern of *Eschericia coli* strains isolated from healthy infants in rural Bangladesh (*n* = 110 isolates)

Class	Antibiotic	Classification of Isolates (%)		
		Sensitive	Intermediate	Resistant
Macrolides	Azithromycin	36.4	0.0	63.6
	Erythromycin	0.0	1.8	98.2
Fluoroquinolones	Ciprofloxacin	78.2	4.5	17.3
	Levofloxacin	80.9	0.9	18.2
Penicillins	Amoxicillin/Clavulanate	75.5	11.8	12.7
	Ampicillin	23.6	6.4	70.0
Cephalosporins	Ceftriaxone	63.6	2.7	33.6
	Cefepime	65.5	10.0	24.5
	Cefuroxime	11.8	54.5	33.6
	Ceftazidime	77.3	12.7	10.0
Carbapenems	Imipenem	87.3	10.9	1.8
	Meropenem	98.2	1.8	0.0
Tetracyclines	Tetracycline	69.1	0.0	30.9
	Doxycycline	75.5	10.9	13.6
Sulfonamide	Trimethoprim/sulfamethoxazole	62.7	3.6	33.6
Monobactam	Aztreonam	68.2	10.0	21.8
Aminoglycoside	Gentamycin	99.1	0.0	0.9
Novel drug	Fosfomycin	100.0	0.0	0.0
Glycylcycline	Tigecycline	97.3	0.9	1.8
Amdinocillin	Mecillinam	97.3	1.8	0.9

Based on cutoffs published by the Clinical and Laboratory Standards Institute.^12^

## DISCUSSION

In this study, we report on resistance patterns in commensal *E. coli* isolated from infants in rural Bangladesh. A high proportion of isolates were resistant to older antimicrobial agents (azithromycin, ampicillin, and erythromycin). The patterns of resistance were similar to those observed in commensal *E. coli* isolated from infants and young children in community settings in rural India, Nepal, Vietnam, and Tanzania^2^^–^^5^ but did not reach the level of antibiotic resistance (95%) reported among healthy infants and children aged 6–72 months in rural Peru and Bolivia.^15^ We observed a higher rate of resistance to cephalosporins (10–33.6%) than has been previously reported in studies conducted with Tunisian children (6.6%)^16^ and Tanzanian children (∼2%).^5^ This may be due to the rapid increase in the prevalence of antibiotic resistance in Bangladesh^17^ relative to countries in Africa.

Carbapenems, along with antibiotics classified as aminoglycosides, novel drugs, glycylcyclines, and amdinocillin, were the most effective among the antibiotics we tested. Carbapenem resistance was low, with only ∼2% of isolates showing resistance to imipenem. Similar results have been reported from testing *E. coli* isolates in Indian children.^18^ This study detected low, but some, resistance to tigecycline (1.8%), which is typically used as a last resort for infections caused by MDR and extensively drug-resistant Gram-negative bacteria. This low percentage of resistance to tigecycline was also reported in a study from India.^2^

Half of the isolates were MDR. A similarly high prevalence of MDR *E. coli* (49.0%) was reported in young children in rural India.^2^ One study conducted among infants in rural Bangladesh reported that almost 77% of the third-generation cephalosporin-resistant *E. coli* were MDR.^19^ Another study reported a fecal carriage rate of 37% MDR *E. coli* among community children in southern Taiwan.^20^ A study from Tanzania also noted a high prevalence of MDR *E. coli* in young children in rural areas.^5^

The strengths of this study include the community-based setting characteristic of rural Bangladesh, which may provide insights into the background prevalence of antibiotic resistance. We also tested a broad panel of antibiotics. However, our microbiological testing was limited to a relatively small subset of infants. Additionally, we tested only one *E. coli* colony per infant. It is possible that other isolates from the same sample would have varying resistance profiles. Together, these limitations prevented any meaningful exploration of risk factors for antibiotic resistance in this setting.

## CONCLUSION

Our study showed that infants’ guts serve as a reservoir for *E. coli *resistant to multiple antibiotics, including cephalosporins, which are critical for the treatment of many infectious diseases in humans.^1^ The high prevalence of intestinal carriage of antibiotic-resistant *E. coli*, including MDR, among infants in a community setting in rural Bangladesh is a serious concern. This underscores the need for additional research and interventions to decrease the carriage of drug-resistant *E. coli* and to control the growing antibiotic resistance crisis.

## Supplemental Materials

10.4269/ajtmh.24-0541 Supplemental Materials
