# Supplementary figures and images for: Multidrug-Resistant Escherichia coli Isolated from Stool Samples of Healthy Infants in Rural Bangladeshi Communities
Source: Am J Trop Med Hyg. 2025 May 13;113(1):134–7. doi: 10.4269/ajtmh.24-0541 (PMC12225563; doi:10.4269/ajtmh.24-0541)

**Supplemental Figure 1:** Flow diagram of infants and biospecimens collected

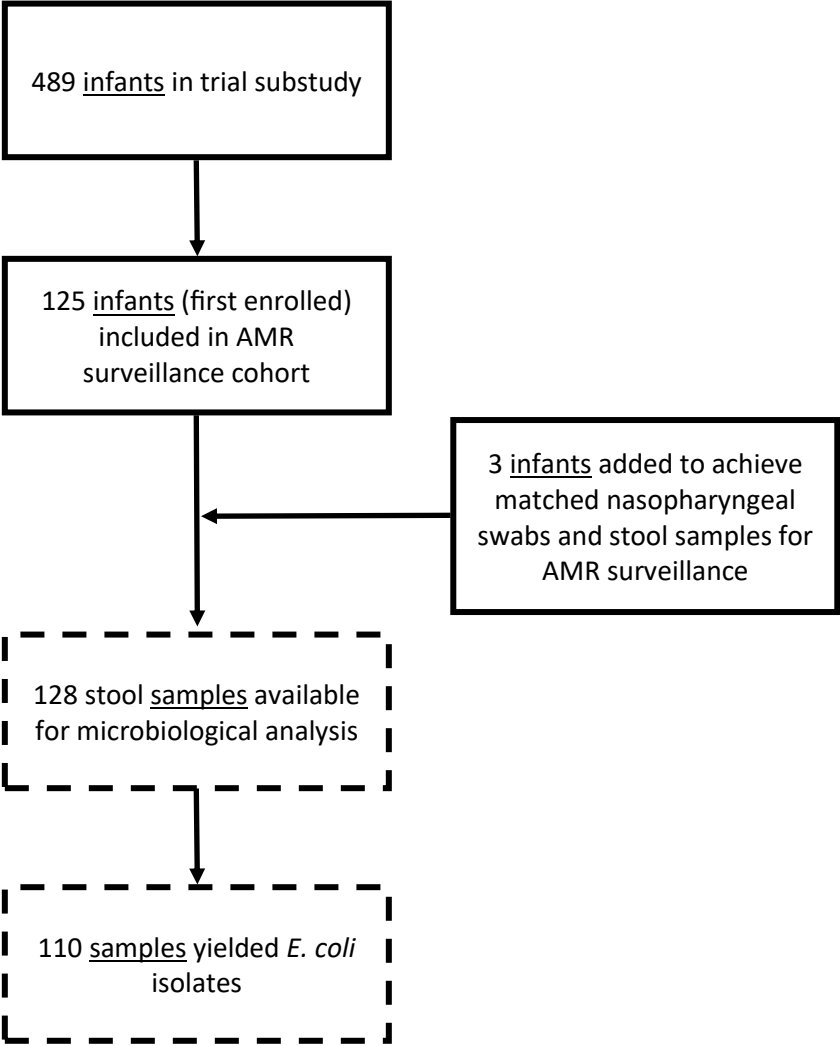

Supplement: Supplemental Materials [file tpmd240541.SD1.pdf]
